# Asymptomatic Carriage of Plasmodium in Urban Dakar: The Risk of Malaria Should Not Be Underestimated

**DOI:** 10.1371/journal.pone.0031100

**Published:** 2012-02-21

**Authors:** Abdoulaye Diallo, Nicaise Tuikue Ndam, Azizath Moussiliou, Stéphanie Dos Santos, Alphousseyni Ndonky, Marion Borderon, Sébastien Oliveau, Richard Lalou, Jean-Yves Le Hesran

**Affiliations:** 1 UMR 216 - Mère et Enfant Face aux Infections Tropicales, Institut de Recherche pour le Développement, Paris, France; 2 Faculté des Sciences Pharmaceutiques, Université Paris Descartes - Sorbonne Paris Cité, Paris, France; 3 UMR151 - Laboratoire Population Environnement et Développement, Institut de Recherche pour le Développement - Université Cheikh Anta Diop, Dakar, Sénégal; 4 UMR151 - Laboratoire Population Environnement et Développement, Institut de Recherche pour le Développement - Université de Provence, Marseille, France; 5 UMR 216 - Mère et Enfant Face aux Infections Tropicales, Institut de Recherche pour le Développement, Cotonou, Bénin; 6 UMR 6012 ESPACE, UFR des Sciences Géographiques et de l'Aménagement - Université de Provence, Aix en Provence, France; Universidade Federal de Minas Gerais, Brazil

## Abstract

**Introduction:**

The objective of this study was to measure the rate of asymptomatic carriage of plasmodium in the Dakar region two years after the implementation of new strategies in clinical malaria management.

**Methodology:**

Between October and December 2008, 2952 households selected in 50 sites of Dakar area, were visited for interviews and blood sampling. Giemsa-stained thick blood smears (TBS) were performed for microscopy in asymptomatic adult women and children aged 2 to 10 years. To ensure the quality of the microscopy, we performed a polymerase chain reaction (PCR) with real time qPCR in all positive TBS by microscopy and in a sample of negative TBS and filter paper blood spots.

**Results:**

The analysis has concerned 2427 women and 2231 children. The mean age of the women was 35.6 years. The mean age of the children was 5.4 years. The parasite prevalence was 2.01% (49/2427) in women and 2.15% (48/2231) in children. Parasite prevalence varied from one study site to another, ranging from 0 to 7.41%. In multivariate analysis, reporting a malaria episode in 2008 was associated with plasmodium carriage (OR = 2.57, P = 0.002) in women; in children, a malaria episode (OR = 6.19, P<0.001) and a travel out of Dakar during last 3 months (OR = 2.27, P = 0.023) were associated with plasmodium carriage.

Among the positive TBS, 95.8% (93/97) were positive by plasmodium PCR. Among the negative TBS, 13.9% (41/293) were positive by PCR. In blood spots, 15.2% (76/500) were positive by PCR. We estimated at 16.5% the parasite prevalence if PCR were performed in 4658 TBS.

**Conclusion:**

Parasite prevalence in Dakar area seemed to be higher than the rate found by microscopy. PCR may be the best tool for measuring plasmodium prevalence in the context of low transmission. Environmental conditions play a major role in the heterogeneity of parasite prevalence within sites.

## Introduction

Malaria is reported to be more prevalent in rural areas compared to urban settings [1], [2]. It is well known that urbanisation is highly associated with the decline of malaria transmission [3]. Indeed, many studies reported little or no malaria transmission in major African cities, as urban settings are less favourable for vector breeding sites [4], [5].

However, despite low transmission, malaria is still a major public health problem in urban areas, as it currently concerns more than 50% of African population and is expected to concern at least 60% in 2050 [6]. Moreover, most studies are quite old, and little is known about the effects of transmission patterns on disease burdens in urban areas [7]. Indeed, urban areas are constantly changing. Urban population growth has outpaced urban planning and sanitation infrastructures. Several problems can be related to rapid spontaneous urbanisation: poor housing, lack of sanitation, lack of drainage of surface water, occupation of the lowlands for housing, practice of urban agriculture. The consequence of this phenomenon is that, in Dakar, for example, several districts are experiencing floods during rainy seasons.

In Senegal in 2005, the Health Ministry reported high levels of malarial diagnoses and anti-malarial prescriptions [8]. However, even if health facilities are present in the cities, they harbour poor populations, and self-medication is common in the general population [9]. Therefore, the assessment of the malaria burden based only on health services could be biased due to missed cases of malaria.

With regard to entomological data, in the south and central sanitary districts of Dakar in 1994–95 and 1996–97 respectively, *Anopheles arabiensis* aggressiveness was low, and no infected *Anopheles* was collected [10], [11]. In the central area, a parasite prevalence of 1% and an annual incidence of clinical attacks of 2.4% have been recorded. However, during 2005–2006, malaria transmission was assessed in two vegetated areas of downtown Dakar; the annual entomological inoculation rate (EIR) was up to 9.5 infective bites per year [12], suggesting a change in the level of malaria transmission in this area.

Available prevalence data for the parasite are out-dated. The city has changed considerably since the last studies on the prevalence of malaria, both demographically and in terms of urbanisation, and it is necessary to obtain useful information to demonstrate the burden of malaria in Dakar.

In 2008, a three-year multi-disciplinary research program was undertaken in Dakar to identify determinant factors of health care access and the success of new strategies in malaria control at the population level.

As a part of this program, the aim of the present work is to update the prevalence of asymptomatic carriage of plasmodium in the Dakar region two years after the implementation of new strategies in clinical malaria management (i.e use of artemisinin-based combination therapy since 2006 and rapid diagnostic tests since 2007), and obtain reliable and representative data.

## Methods

### Study area

The study took place in the region of Dakar, Senegal, which comprises the *districts* of Dakar, Pikine, Guediawaye and Rufisque.

The *districts* are sub-divided into 14 boroughs and 42 *neighbourhoods* (akin to boroughs).

In 2002, these 4 urban *districts* had a population of 2,168,314 inhabitants and 302,551 households [13]. This population represents 22% of the total Senegalese population and lives in only 0.3% of the total surface area of the country. Based on a 2.5% annual growth rate, the population in the Dakar region was estimated to be 2,493,561 in 2008. Housing types are residential, planned, spontaneous legal and spontaneous illegal (shanty towns). In the region of Dakar, spontaneous housing represents more than 30% of inhabited areas. The rate of illegal housing is estimated to be 21.7% in the entire region, with 2.9% for the district of Dakar, 42.4% for Pikine and 9.5% for Rufisque.

The region of Dakar is in a Sudano-Sahelian climate with a long dry season (October to June) and a short rainy season (July to September). The level of recorded rainfall rarely exceeds 500 mm per annum. However, it has been increasing since 2005 [14], reaching 510 mm in 2008; most of this quantity fell in one week, causing major floods in the suburban areas of Dakar.

Due to the proximity of the sea, this coastal area benefits from particularly mild conditions, with a maximum temperature of 27–28°C in September and a minimum of 21–22°C in February.

### Study type and population

We performed a cross-sectional survey from October to December 2008.

Following the model of household surveys using the IRIS (Grouped Islets for Statistic Indicators) carried out within the SIRS research program (health, inequalities and social breakdown in France) [15], 3,000 households should be visited.

The eligible households must contain at least one child younger than ten.

### Selection of study sites

The urban environment is known to be a heterogeneous space from both a socio-economic and an environmental perspective. The aim of the choice of study sites was therefore to highlight the diversity of the urban zone. It was necessary to have homogeneous zones and the most heterogeneous zones between them.

The “*district de recensement*” (DR - or census area) given by the national agency of statistics and demography [13] provides a way of meeting this objective. Indeed, the DR is the most refined level of census data **collection**. The population average in each DR was 1037 inhabitants, 141 households and 86 concessions. One DR constituted a homogeneous entity from a socio-economic, demographic and environmental point of view. Dakar comprises approximately 2000 DR.

The selection of study sites followed a 3-step procedure, described below.

#### Reduction of census data

Census data are too bulky to be processed directly. Data synthesis is thus necessary. We first regrouped various clusters of variables belonging to the same topic (housing, household equipment, sanitation). For each group, a principal component analysis (PCA) was performed to extract the first two factorisation axes summarising the data for each group.

Finally, a principal component analysis was carried out across all factors to perform a factorial analysis.

#### Classification of DR

A classification by dynamic clusters (K-means method) using variables selected from the PCA enabled us to identify 5 types of DR.

**Type 1:** 307 DR, located mostly in the district of Dakar. This type mainly consists of the high-income population, which enjoys good housing and living conditions.

**Type 2:** 274 DR, located mostly in the district of Dakar. This type mainly consists of the middle-income population.

**Type 3:** 335 DR can be found in clusters in various areas. This type contains the low-income populations, mainly in the district of Dakar.

**Type 4:** 528 DR can be found in clusters across various built-up areas, especially to the west of Pikine. This type mainly contains low-income populations from the old Pikine.

**Type 5:** 526 DR found in clusters across various built-up areas, especially in the east Dakar area. It is mostly composed of the low-income populations from recently occupied zones found mainly to the east of Pikine.

#### Selection of the study areas, the households and the individuals

The most representative types in each *neighbourhood* were chosen. Then, 42 DR were randomly chosen. To reach the number of 50 sites planed for the study, 8 other DR were then added according their socio-demographic and economic characteristics as well as to their proximity to the swamps. To avoid the risk of insufficient numbers of households in a DR, we have paired each of the chosen DR with the nearest same type of DR, thereby constituting one study site.

Selected DR were visited according to itineraries pre-established by the head supervisor; the field worker picked up a concession within the study zone and then moved from one household to the next. If a concession encompassed several households, the field worker chose the head of the household using the first name ranked alphabetically.

Sixty households in each site (3,000 households for the 50 sites) should be selected. The first criterion for household selection was the presence of at least one 2- to 10-year-old child. After collecting a family agreement, socio-demographic investigators completed a questionnaire about the household lifestyle, education level, income, and the access mode to healthcare facilities. The adult woman (generally the child's mother) who answered the questionnaire and a child aged between 2 and 10 years old were selected by the nurses for blood sampling. Asymptomatic individuals were selected on the basis of their own declarations and interviews by nurses. Pregnant women and individuals who were sick and/or had taken anti-malarial drugs during the 15 days preceding the blood sampling were not included in the study. Then a second questionnaire was completed by a team of nurses, exploring events linked to a reported malarial attack, including the history of a malarial episode during 2008, the use of a bed net, any overnight stays outside the region of Dakar (for women), or any travel (children).

Reporting a malaria episode in 2008: we asked individuals (women and caretakers for children) if they have experienced a malaria attack during the year 2008.

### Ethics Statement

This protocol was approved by the ethics committee of Senegal's Department for Health (SEN 12/08). Information about the objective of the study was given to the head of the household, and verbal consent was requested. For minors, the consent of their legal guardians was requested. This procedure was used because most of the head of household could not write. The nurse explained the aims and interest of the study, and then he signed a document proving that he has obtained the consent. The ethics committee agreed on this procedure.

All participants were informed of the results of the thick blood smear examination by telephone or home visits. Those who tested positive were treated through the local health facility.

### Biological measures

A thick blood smear for malaria parasite research and a dried blood filter paper spot were collected from each volunteer. The staining and the reading of the thick smears were carried out at the laboratory of the research institute for development (IRD) in Dakar.

To declare that a thick smear is negative, the microscopist examined at least 200 oil immersion fields. A control examination of all thick smears that were found to be positive was performed. Ten percent of the negative thick smears have been examined a second time to guarantee the quality of the reading. All discrepancies were discussed, and if necessary, the thick smears were read by a third microscopist. The plasmodium species and the presence or absence of gametocytes was noted. We counted the number of trophozoites per 200 leucocytes. If the number of trophozoites counted is less than 10 per 200 leucocytes, the count is made from 500 leucocytes. We calculated the parasite density per microliter of blood using the following formula: (Number of trophozoites ×8000)/Number of leucocytes

### Polymerase Chain Reaction (PCR) procedures

#### DNA extraction

DNA was extracted from filter spots by the use of chelex as previously described [16]. DNA extraction from archival Giemsa-stained blood smear slides was carried out according to the Qiagen method described by Cnops et al. [17] with minor modifications. Briefly, the Giemsa-stained TBF was scraped off the slide with a sterile scalpel and collected into phosphate-buffered saline. DNA was extracted from the collected material with the Qiagen DNeasy Blood and Tissue Kit (Qiagen, France) according to the manufacturer's instructions. DNA was eluted in 100 µL of elution buffer (provided with the kit).

#### Real-time PCR

Duplex real-time PCR was performed using the conditions 95°C for 5 s and 60°C for 45 seconds with the Rotor Gene 6000 analytical PCR system. The PCR reaction was performed in a single reaction tube with a final volume of 20 µl, containing 5 µl of DNA, 10 µl of PerfeCTa qPCR FastMix, UNG (Quanta Biosciences), 300 nM of each primer, and 100 nM of each probe. We used the genus-specific primers Plasmo1 (GTT AAG GGA GTG AAG ACG ATC AGA) and Plasmo2 (AAC CCA AAG ACT TTG ATT TCT CAT AA) and the *Plasmodium falciparum*-specific forward primer Fal-F (CCG ACT AGG TGT TGG ATG AAA GTG TTA A) designed from sequences of the small subunit of 18S rRNA and reported by Shokoples et al. [18]. Minor sequence modifications were made to the probes used by Shokoples et al. to increase specificity and cross hybridisation of primers and probes in the duplex: we used minor groove binder (MGB) probes for each primer pair (PlasmoProbe: Vic-TCGTAATCTTAACCATAAAC and Falciprobe: Fam - TCTAAAAGTCACCTCGAAAGA) to increase specificity and limit cross-hybridisation of primers and probes in duplexes. Forty cycles of PCR were carried out and included the following controls: extractions of PBS and/or chelex as a negative control, a human host gene (human glyceraldehyde-3-phosphate dehydrogenase (GAPDH)) as a positive control for DNA extraction using primers and probe described in [19], 3D7 DNA isolated from a slide or a filter spot as a reference positive control, and a no-template control. All oligonucleotides were synthesized by Applied Biosystems (Courtaboeuf, France).

### Statistical analysis

Statistical analysis was performed using Stata version 11 (www.stata.com). The socio-demographic characteristics (age, sex for children and education level for adult women) were described.

Three malariometric indices were calculated (microscopy results):

- Parasite prevalence: the percentage of thick blood smears carrying a plasmodium.
- Gametocyte index: the percentage of positive thick blood smears carrying a gametocyte.
- The parasite density (DP): e.g., parasitaemia. The parasite density was estimated in relation to leucocytes, on the basis of 8,000 leucocytes per µl of blood, as explained above.

The parasite prevalence was calculated for two groups, women and children, in each study site.

The χ2 test was used to compare the plasmodium carriage rate in women and children.

To estimate the association between the presence of plasmodium and the variables mentioned above, a univariate analysis by logistic regression was performed first. The ages in the women's group were divided into 4 classes based on quartiles. In children, two age classes were created (<5 years and ≥5 years). Finally, a multivariate logistic regression was performed to test the association between plasmodium carriage and independent covariates.

A test was considered statistically significant if the p-value was <0.05.

## Results

Two thousand nine hundred and fifty-two households in 50 study sites were visited for both socio-demographic and biological investigations. Of these, 328 households did not give consent for biological measures, and 63 households were missing or unavailable at the time of the home visit. Other subjects were not selected due to protocol requirements (i.e., illness, pregnancy). The analysis of biological data has finally concerned 2427 women and 2231 children.

The mean age of the women was 35.6 years (range = 17–81). The mean age of the children was 5.4 years (range = 2–10). Forty-nine percent of the children were male.

### Parasite prevalence by microscopy

The parasite prevalence was 2.01% (49/2427) in women and 2.15% (48/2231) in children, while the overall parasite prevalence was 2.08% (97/4658).

A total of 96.9% of infections were caused by *Plasmodium falciparum*; two thick smears (2.06%) harboured *Plasmodium malariae*, while one individual had a mixed infection (*Plasmodium falciparum* and *Plasmodium malariae*).

Gametocytes were found in 33 individuals among the total of 97 infected by plasmodium (34.02%): 34.7% (17) in women and 33.3% (16) in children. Gametocyte carriage was higher among children younger than 5 (50% vs. 16.7%, p = 0.030).

The parasite density (DP) for asexual forms ranged from 1.1 to 41963 parasites per microliter, with a median value of 190. Among positive thick blood smears, 25% had parasitaemia of less than 12 parasites per microliter of blood.

The overall parasite prevalence did not show monthly variations. It was at 2.10%, 2.15% and 1.95% in October, November and December, respectively.

### Prevalence of infection by age (microscopy results)

In women, the plasmodium prevalence was 2.93% in the population younger than 28 years old, compared to 1.08%, 1.96% and 1.91% for the age classes of 28–34, 34–42 and more than 42 years, respectively. The difference was not significant.

In children, there was no difference between the group younger than 5 (2.03%) and the group 5 and older (2.29%).

### Prevalence by study site (microscopy results) (Table 1, Figure 1, Figure 2)

**Figure 1:**
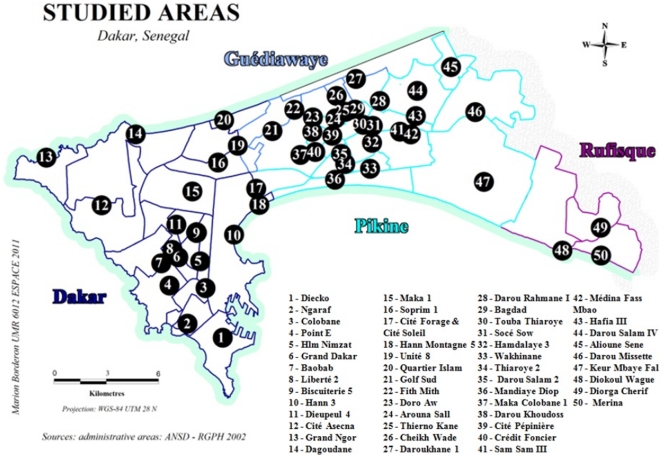
Map of the Dakar region. Black circles indicate the location of 50 study sites in 2008. Names of sites are listed below.

**Figure 2:**
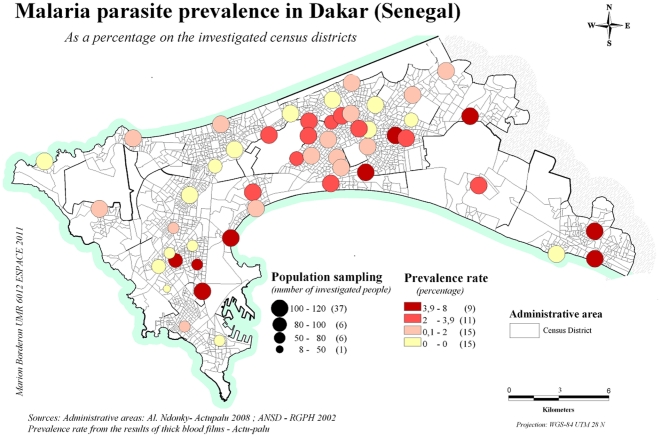
Plasmodium distribution by study site at microscopy (Women and children) in 2008 in Dakar (malaria parasite prevalence is the percentage of positive thick smears in each site).

**Table 1:** Parasite prevalence by study site (only sites where parasites have been found by microscopy are displayed in this table). The total represents all the blood thick smears readed in the 50 sites (Dakar 2008).

	Women	Children			
Study sites	(No of positive) N1	(No of positive) N2	Total positive	Sample size	Prevalence (95%IC)
Ngaraf	(0)26	(1)22	1	48	2.08% (0–6,27)
Colobane	(1)49	(3)48	4	97	4.12% (0–8,15)
Grand Dakar	(3)44	(1)41	4	85	4.71% (0,11–9,30)
HLM Nimzat	(3)35	(1)30	4	65	6.15% (0,15–12,15)
Hann Montagne 5	(1)55	(0)52	1	107	0.93% (0–2,78)
Hann 3	(2)50	(2)49	4	99	4.04% (0–7,98)
Dieupeul 4	(0)35	(1)29	1	64	1.56% (0–4,68)
Cité ASECNA	(1)51	(1)46	2	97	2.06% (0–4,94)
Dagoudane (Yoff)	(0)55	(2)53	2	108	1.85% (0–4,43)
Islam	(1)50	(0)47	1	97	1.03% (0–3,07)
Golf Sud	(2)52	(1)44	3	96	3.13% (0–6,66)
Doro Aw	(2)50	(1)44	3	94	3.19% (0–6,81)
Daroukhane 1	(0)51	(2)51	2	102	1.96% (0–4,69)
Arouna Sall	(1)51	(1)41	2	92	2.17% (0–5,21)
Thierno Kane	(3)55	(1)54	4	109	3.67% (0,08–7,25)
Darou Salam 4	(1)53	(0)47	1	100	1.00% (0–2,98)
Alioune Sène	(1)52	(1)54	2	106	1.89% (0–4,51)
Darou missette	(6)56	(2)52	8	108	7.41% (2,38–12,42)
Maka Colobane 1	(2)48	(0)48	2	96	2.08% (0–4,99)
Crédit Foncier	(0)53	(1)54	1	107	0.93% (0–2,78)
Cité Pépiniére	(0)47	(1)52	1	99	1.01% (0–3,01)
Darou Khoudoss	(1)50	(2)44	3	94	3.19% (0–6,81)
Dalifort	(1)49	(2)37	3	86	3.49% (0–7,44)
Bagdad	(1)56	(1)52	2	108	1.85% (0–4,43)
Touba Thiaroye	(2)57	(1)56	3	113	2.65% (0–5,66)
Darou Salam 2	(1)51	(0)49	1	100	1.00% (0–2,98)
Thiaroye 2 (Sotrac 1)	(1)51	(1)47	2	98	2.04% (0–4,89)
Mandiaye Diop	(2)51	(1)52	3	103	2.91% (0–6,21)
Wakhinane	(1)55	(4)48	5	103	4.85% (0,63–9,07)
Sam Sam 3	(2)56	(3)51	5	107	4.67% (0,6–8,73)
Medina Fass Mbao	(1)52	(2)47	3	99	3.03% (0–6,46)
Hamdalaye 3	(0)52	(2)52	2	104	1.92% (0–4,6)
Keur Mbaye Fall	(0)57	(3)52	3	109	2.75% (0–5,87)
Diorga Chérif	(2)57	(3)53	5	110	4.55% (0,59–8,49)
Mérina	(3)56	(1)47	4	103	3.88% (0,08–7,67)
**Total**	(49)2427	(48)2231	(97)	4658	2.08% (1,67–2,49)

N1, N2: number of thick smears examined by microscopy. No: number.

Parasite prevalence varied from one study site to another, ranging from 0 to 7.41%. No plasmodium carriers were found in fifteen sites (Figure 2).

In the women, the parasite prevalence ranged from 0 to 10.71%, whereas in children, it ranged from 0 to 8.33%. Differences of parasite prevalence are found throughout the area of Dakar. Differences are more pronounced in peripheral sites located nearest the swamps. A chi-square fisher exact test, have been performed to compare prevalence between sites. For example, prevalence in Darou missette is 7.41 whereas it is 1% in Darou salam 2 (p = 0.036). Similarly, prevalence is 0% for Diokoul and 3.88% for Merina (p = 0.053).

Three sites closest to the downtown of Dakar (Colobane, Grand Dakar and HLM Nimzatt) had parasite prevalences as high as those found in peripheral areas.

### Risk factors for infection in women (microscopy results) (Table 2)

**Table 2:** Risk factors for plasmodium carriage in women (logistic regression using microscopy results, Dakar 2008).

			Univariate analysis		Multivariate (final model)	
Covariates	Class	Positive(N)	OR**	P***	OR**	P***
Age (years)	< = 28	20(682)	1	0.136	1	
	28–34	6(555)	0.36		0.39	0.053
	34–42	12(613)	0.66		0.66	0.296
	>42	11(577)	0.64		0.71	0.397
Education level	None	21(973)	1	0.57	*	
	Primary	18(867)	0.96			
	Secondary	6(437)	0.63			
Malaria episode in 2008	Yes	27(747)	2.82	<10^−3^	2.57	0.002
	No	22(1680)	1		1	
Bed net	Yes	25(1039)	1.27	0.396	*	
	No	24(1268)	1			
Night out of Dakar	Yes	7(556)	1	0.168	1	
	No	38(1721)	1.77		1.73	0.184

*: covariates not introduced in the final model, OR.

**: Odds Ratio, P.

***: p-value, N: Sample size in each class.

In the univariate analysis, only “reporting a malaria episode in 2008” was significantly associated with parasite carriage. Among women, 45% declared using a bed net; we have found no differences in parasite prevalence between women who reported using a bed net and those who did not.

In the multivariate analysis, only age and “reporting a malaria episode in 2008” were significantly associated with parasite carriage. Women between 28 and 34 years were less likely to be infected compared to those aged younger than 28 (OR = 0.37, p = 0.05).

A Hosmer-Lemeshow test was performed to verify the quality of model fit. The results of the test (X ^2^ = 4.28, p = 0.74) confirms the good fit of the model.

### Risk factors associated with malaria infection in children by microscopy (Table 3)

**Table 3:** Risk factors for plasmodium carriage in children (logistic regression using microscopy results, Dakar 2008).

			Univariate analysis		Multivariate (final model)	
Covariates	Class	Positive(N)	OR**	P***	OR**	P***
Sex	Male	24(1090)	1		1	
	Female	24(1141)	0.95	0.873	1.03	0.918
Age (years)	<5	24(1182)	1		1	
	> = 5	24(1045)	1.13	0.666	1.02	0.95
Malaria episode in 2008	Yes	33(824)	3.87	<10^−3^	6.19	<10^−3^
	No	15(1403)	1		1	
Bed net	Yes	20(922)	1.19	0.571	*	
	No	22(1206)	1			
Travel	Yes	11(304)	2.18	0.029	2.27	0.023
	No	31(1832)	1		1	

*: covariates not introduced in the final model, OR.

**: Odds Ratio, P.

***: p-value, N: Sample size in each class.

In the univariate analysis, “a malaria episode in 2008” (OR = 3.87, p<0.001) and “a trip out of Dakar in last three months “ (OR = 2.18, p = 0.029) were significantly associated with parasite carriage. We did not find any differences in risk according to age group or sex.

In children, bed net use as reported by mothers was 43%; no difference in prevalence was found according to bed net use.

In the multivariate analysis, “a malaria episode in 2008” and “a trip out of Dakar in 2008 in last three months” were associated with parasite carriage, with an OR of 6.19 (P<0.001) and 2.27 (p = 0.023), respectively.

A Hosmer-Lemeshow test was performed (X ^2^ = 6.62, p = 0.46) and confirmed the hypothesis of equality between the observed and the predicted number.

### PCR results (Table 4)

**Table 4:** Results of polymerase chain reaction (Dakar 2008).

	Microscopy	Positive by PCR	Percentage
Thick smears	Positive(97)	93	95.8%
	Negative(293)	41	13.9%
Blood spots	Negative(500)	76	15.2%

PCRs were subsequently carried out on DNA extracted from archival Giemsa-stained thick smears or blood spots to ensure the quality of the microscopy readings. Ninety-seven positive thick smears, 293 negative thick smears, and 500 dried blood spots corresponding to negative thick smears were randomly selected. The 3D7 positive control reproducibly amplified appropriately with pan-species and *Plasmodium falciparum*-specific primers and all negative controls failed to amplify. In all samples, the human GAPDH gene amplified appropriately, confirming successful extraction of DNA from both the filter papers and thick smears.

Among the positive thick smears, 95.8% (93/97) were positive by plasmodium PCR. Among the negative thick smears, 13.9% (41/293) were positive by PCR. In the dried blood spots, 15.2% (76/500) were positive by PCR.

To calculate the overall parasite prevalence, we first calculated the total percentage of positive PCR results in a selected sub-sample; 41 of 293 thick smears and 76 of 500 blood spots were positive, giving an overall rate of 14.75%.

Second, we estimated 673 positive thick smears if PCR was performed on 4561 negative thick smears (97 positive thick smears removed) using the rate of 14.75% found above in the sub-sample.

Based on these results, the estimated parasite prevalence using PCR and microscopy is approximately 16.5% in our sample (673 positives estimated by PCR added to 97 positives by microscopy, from a total of 4658 thick smears).

## Discussion

### Current parasite prevalence/previous parasite prevalence

The aim of this study was to measure parasite prevalence in a sample of asymptomatic individuals in the region of Dakar. The selection of study sites and households was performed by following a methodology that takes in account the diversity of the urban environment. The representativity of the sample was also established with regard to population density. Data were obtained from the 2002 population census provided by the national agency for statistics and demography of Senegal. During the period 2002–2008, there was a small increase in population density; indeed, Dakar was urbanised at 97.2% in 2002. Moreover, Dakar is built on a peninsula, which allows possible extensions only on the east side of the area. Changes have mostly concerned the newly occupied areas in the east of Dakar, where only 3 study sites were selected in our sample.

Our study was mainly focused on women and children aged 2 to 10 years old. It was easier for us to interview women, who are responsible for the daily activities of the home. Men are often missing due to work or travel. Children aged 2 to 10 constitute a standard group for studies of malaria endemicity, but measures in women could present a bias in the estimation of parasite prevalence in the adult population. No study has demonstrated that women have a higher risk of carrying the parasite than male adults in Dakar. Nevertheless, to obtain an indication of the prevalence in men, 224 thick smears were collected in volunteer men present at home during our visit. Five of them were found to be positive (2.23%). This prevalence is similar to that found in women.

Several population studies have been conducted in Dakar area since 1979. Four (4) main studies were still references for malaria transmission in Dakar. Two studies conducted in 1979 and 1987 in Pikine located at 15 kilometers away of downtown of Dakar; the target population were children aged 6 months to 6 years in the first study [20] and all-age population [21] in the second study.

Between 1995 and 1997, Diallo et al. [22], [11] conducted two surveys in and near the downtown of Dakar in general population. In all studies, thick blood smears were used to detect plasmodium.

In Pikine parasite prevalence were 8.8% in 1980 and 3.8% in 1988 respectively, while in our survey, parasite prevalence varied from 0.88 to 3.88% in the sites located near these areas. Thus, there is variation in parasite prevalence, mostly explained by increasing urbanisation, since Pikine was not highly urbanised in the early 80 s.

Parasite prevalence in Diallo studies, were 0.9% (October–November 1994) and 1.5% (October–November 1996) in downtown Dakar and the nearby respectively, whereas in our study, it was 2.08%, 4.12% and 6.1% for the sites located at nearby. Accordingly, prevalence is higher in our study.

The methodologies in all these studies are different in terms of area and target population.

In our sample, children under 5 years old have a carriage rate of 2.03%. This rate is higher than the 0.8% found recently by the ENPS (National Survey for Malaria in Senegal) in infants aged 6 months to 59 months in 2008 in Dakar [23].

Thus, the variability of parasite prevalence across time and space is evident in Dakar.

### Prevalence estimated by PCR

The same technique was used in all studies mentioned above, i.e., thick blood smears (BS). We used real-time qPCR to confirm our microscopy results. Unfortunately, we were unable to carry out PCR on all the blood samples, given the high cost of the technique. Several studies have shown the superiority of real-time qPCR over microscopy [24], [25], [26]. Malhotra et al. showed that infection rates among pregnant women attending a Kenyan hospital district ranged from 9.4% by BS to 37.9% by real-time qPCR [27]. Similarly, a study conducted in Mozambique in pregnant women showed a plasmodium prevalence of 5.3% by microscopy and 23.2% by real-time qPCR [28]. In these studies, BS was defined as negative if no parasites were observed in 200 oil immersion fields, the same criterion used in our study.

Nevertheless, in our study, 4 positive BS were negative by PCR; parasitaemia was 19.4, 12.9, 4.8 and 137.1 parasites per microliter. For these BS positive samples that were not detected by PCR, several reasons could explain this: artefacts in thick smear reading due to red cell fragments, other blood elements or environmental contaminant, which might produce false-positive in microscopy. This has been discussed in other studies [29], [30], [19]. On the other hand, the quality of DNA extracted from BS and blood spot can also affect the molecular diagnostics [31]. However, the primers used in this study were adopted from an optimized assay which proved high sensitivity (a lower detection limit of 1 copy per reaction mixture) and specificity [18].

Most clinical and field studies on malaria rely on BS and PCR has not been widely used principally because of poor resources and infrastructures in study areas. The application of microscopy in areas with low parasite prevalence or parasitaemia can compromise its predictive value. The overall prevalence estimated by PCR in our study is approximately 16.5% which is 8-time higher than the microscopy.

If we conclude that malaria remains endemic in Dakar and that the prevalence can reach high levels in some areas (more than 10%), these results could explain the high consumption of anti-malarial drugs that has been found in Dakar, with high self-medication [9], [32]. These data suggest two main public health problems. First, the high PCR or sub-microscopic prevalence of the malaria parasite underlines the necessity of redefining the burden of malaria in urban areas. These infections, even at low densities, could be a potential source of transmission for vectors [33] and a potential source of malaria attack within the population. Secondly, self-diagnosis and consequently self-medication are high in Dakar. People can easily gain access to ACT without prescriptions in drugstores. Thus, the risk of emergence of *Plasmodium falciparum* resistance to new anti-malarial drugs is quite plausible in this context, as artemisinin resistance has been reported recently in Cambodia [34]. In addition, a study conducted in 2004 in Dakar showed that severe malaria in adults is associated with inappropriate self-treatment and mutations in genes; the gene mutations are associated with drug resistance [32].

### Entomological considerations

These results could be related to new entomological data collected in the same areas. Entomological data collected in Dakar before 2000 suggested very low or no malaria transmission, with an EIR of less than 1 infected bite per person per year [21]. An urban environment is not adequate for *Anopheles* development. In urban Dakar, malaria infections were imported from rural villages. However, recent data suggest that transmission occurs in Dakar. Indeed, a two-year study (2005/2006) in two sites near Dakar-centre (Ouakam and Bel-air) showed an annual EIR of 3 and 3 in Ouakam and 9.5 and 4 in Bel-air in 2005 and 2006, respectively [12]. The following year, the same team continued the study in a dozen sites in the region of Dakar, and the annual EIR varied from 0 to 16.8, with a rate of 5.8 in Pikine [35].

The hypothesis is that the mosquito responsible for the transmission of malaria has adapted to urban sites and now can develop in polluted waters, as reported by Awolola et al. in Lagos [36]. In addition, urban agriculture is quite frequent in Dakar, creating wet spaces for mosquitoes, and is the major factor driving resistance to insecticides [37].

We found that approximately one-third (34%) of positive thick smears were gametocyte carriers. This rate rises to 50% in children under 5 compared to 17% for the other children. These findings are similar to those found in other studies [38], [39]. It has been described that gametocytes can persist for up to 6 weeks after complete treatment [40]. Thus, in reference to the PCR results, there may be a huge potential reservoir of parasites in the region of Dakar. This reservoir might be underestimated, thereby sustaining malaria transmission.

### Comparison women/children

In the low transmission context, such as urban areas, the constitution of immunity against malaria is delayed and often incomplete. Therefore, adults and children could face the risk of infection equally in areas with instable transmission. We did not find a significant difference between the prevalence in children (2.15%) and women (2.01%). Adults seem to be vulnerable in urban areas and can present with severe malaria [32], [41]. Our results confirm this situation. The susceptibility of adults to plasmodium infection suggests a non-negligible risk of malaria attack and severe malaria in Dakar. We have no information about the burden of malaria in the public health system.

### Environmental Conditions

Our study has demonstrated that the plasmodium is widely spread in the region of Dakar. Indeed, among the 50 sites surveyed, an absence of parasites was found by microscopy in only 15 areas.

The built-up area of Dakar presents a diversified environment, with varying levels of sanitation from one zone to another. The city centre and surrounding districts are well urbanised. In contrast, the peripheral zones, which include the marshlands (interdunar depressions, mostly humid and suitable for gardens), are characterised by a mix of urbanised neighbourhoods and those occupied illegally.

Depending on the study area, the prevalence varied from 0 to 10.8% in women and from 0 to 8% in children. Similarly, we observed huge variations in neighbouring sites. Three sites (Colobane, Grand-Dakar and HLM Nimzatt) located downtown Dakar presented a rather high prevalence. This could be explained by local characteristics that must be explored by geographical and entomological studies. The other sites located downtown Dakar presented rather low levels of parasite prevalence. In contrast, parasite prevalence is high at sites located in peripheral areas. Indeed, these areas presented 2 characteristics: first, urban farming is developing near the swamps (“Niayes”), and second, illegal dwelling has modified local conditions, causing major floods during rainfall.

In the present study, we found differences in parasite prevalence between sites; but it was not possible to demonstrate the heterogeneity of prevalence. However the differences observed between sites suggest strongly the existence of the heterogeneity of malaria transmission, as it is showed in an entomological study conducted between 2007 and 2010 in Dakar [42].

Environmental factors play an important role in relation to the risk of infection. People living near a potential larval habitat are more at risk of infection than others [43].

### Risk factors

#### Bed nets

The use of bed nets is known to reduce the risk of malarial infection. In this study, self-reported bed net use was 45% for women and 43% for children. These rates do not differ from those found in the National Survey for Malaria in Senegal [23], with 41.4% for Dakar, which is significantly lower than the rate found in rural areas by the same survey (78.7%).

Surprisingly, we have found no significant difference in the prevalence of malaria between those who used a bed net and those who did not. Data were collected by interview. These results show the limitation of self-reported information. Indeed, if individuals have a bed net, this does not mean that they use it correctly and consistently. To establish any link between malaria infection and the use of bed nets in the general population, it would be necessary to perform a questionnaire on habits and day-to-day usage.

#### Malaria attack in 2008

The period of the survey coincided with the maximum period of malaria transmission in Dakar [44]. We were only interested in individuals who presented no symptoms and who took no anti-malarial drugs during the 2 weeks preceding blood sampling.

We have found that most children carrying plasmodium have had a clinical attack of malaria (69% of infected children declared a history of malaria during the year 2008 vs. 31% uninfected). The same situation was found in women.

We suggest that the majority of infections occurred recently, since the beginning of rainy season. Most treatments were self-medication, with problems of compliance and adequate treatment, which can explain the lack of parasite clearance. The other hypothesis is that the sites with a high risk of infection were most concerned in our study. This could explain the correlation between a history of malaria and plasmodium carriage. When regrouping the sites with higher prevalence, we found a carriage rate of 7.43% in individuals with a history of malaria and 2.23% for others (X^2^ = 12, p = 0.001). Similarly, by regrouping all sites with a lower prevalence, the carriage rate was 1.85% in individuals who have a history of malaria and 1.15% for others (the difference is not significant).

#### Travelling

In children, plasmodium carriage is significantly associated with travel. No significant risk has been found in women. Many families send their children out of Dakar (often to rural villages) during holidays corresponding with the rainy season. The risk of infection is more important in rural areas, especially if the stay is long.

The movement of this population affects malaria transmission [22]. The role of imported malaria is not negligible for the burden of malaria in urban Dakar. However, new studies are necessary to evaluate the weight of this potential source of infection.

### Conclusion

Our results outline a new urban malaria epidemiology. Contrary to what is commonly accepted, malaria is not uncommon in urban Dakar, and malaria is not unstable. Data suggest that malaria may still be a threat to the urban population. As a confirmation, three facts must be considered. First, entomological data clearly show an increased risk of transmission. Second, our data show a parasitological prevalence that is variable but very significant in certain areas, especially in the most popular part of the town. Third, self-medication in drugstores is frequent and suggests a significant rate of malaria attack in population.

The weight of malaria in common diseases is certainly underestimated due to the huge distribution, mostly un-regulated, of new treatments (ACT), reduced access to care and high rate of self-medication.

Our results and entomological findings should help to initiate further studies in populations and to alert health authorities to take into account environmental conditions. According to these findings, it should be important to update the burden of clinical malaria in the Dakar area, including severe malaria, to set up a monitoring system for severe cases and for screening of the emergence of drug resistance.

Our study also suggests that PCR may be the best tool for the estimation of parasite prevalence in the general population in urban Dakar due to the low rate of parasitaemia occurring in this context.
